# YAP: a novel target for Alzheimer's disease

**DOI:** 10.18632/aging.204045

**Published:** 2022-04-28

**Authors:** Xingxing Xu, Ying Wang, Zhihui Huang

**Affiliations:** 1 School of Basic Medical Sciences, Wenzhou Medical University, Wenzhou, China; 2 College of Pharmacy, Hangzhou Normal University, Hangzhou, China; 3 Clinical Research Center, Affiliated Hangzhou First People’s Hospital, Zhejiang University School of Medicine, Hangzhou, Zhejiang, China

**Keywords:** YAP, Alzheimer's disease, senescence, astrocytes

Alzheimer's disease (AD) is a serious neurodegenerative disease, progressing with aging. Although the well-known amyloid β (Aβ) theory and hyperphosphorylation of tau protein caused neurofibrillary tangles have been uncovered to be the critical pathogenesis in AD, no effective treatment is available so far, and the mechanisms underlying AD are not completely understood.

Emerging evidence has shown that senescent astrocytes is involved in initiating and promoting the progression of AD [1]. Senescent astrocytes show large, flat, and vacuolated cell morphology, increased senescence-associated β-galactosidase activity, cell cycle arrest, up-regulation of p16, p53 and p21, down-regulation of Lamin B1, and expression of senescence-associated secretory phenotype (SASP) [1]. It has been reported that the number of senescent astrocytes in the frontal cortex of AD patients is significantly higher than that of non-AD adults with similar ages and fetal controls [2,3]. Accumulation of senescent astrocytes leads to massive secretion of SASP factors, reduces Aβ clearance, promotes aggregation of insoluble tau. Elimination of these senescent glial cells, including astrocytes, prevents the hyperphosphorylation of tau protein, neurofibrillary tangles, and cognitive hypofunction [4]. Interestingly, Aβ application produces classical phenotypes of senescence in human astrocytes *in vitro*, suggesting complex interaction between astrocytic senescence and Aβ deposition [2]. Therefore, astrocytic senescence is a component of AD, and may be a novel contributor to pathogenesis in AD. However, the mechanisms underlying the senescence of astrocytes in AD remain unknown.

**Figure 1:**
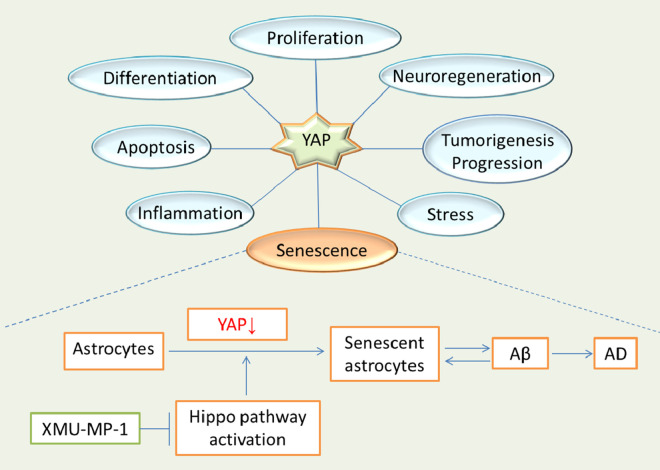
**The new role of YAP in astrocytic senescence and AD.** YAP is a protein that participates in various physiological and pathological processes, and functions in cell proliferation, differentiation, apoptosis, inflammation, neuroregeneration, tumorigenesis and progression, and stress. Moreover, YAP plays an important role in cellular senescence as well. Down-regulation of YAP promotes senescence of astrocytes, and may increase deposition of Aβ, which in turn, aggravates senescence of astrocytes, and such complex interaction contribute to AD pathogenesis.

Yes-associated protein (YAP), as a co-activator and multi-functional protein, is a critical effector of the Hippo pathway, and has been shown to inhibit the senescence of various types of cells, such as glioblastoma cells [5]. YAP is down-regulated in D-galactose-induced senescent glioblastoma cells, and over-expression of YAP partially reverses the senescence of glioblastoma cells, indicating a role of YAP in cellular senescence [5]. Recently, we have found that YAP is down-regulated and inactivated in senescent astrocytes, not only in cultured senescent astrocytes, but also in hippocampal astrocytes of the aging mice and AD model mice, in a Hippo pathway-dependent manner, indicating a role of YAP in astrocytic senescence [6]. Selectively knockout YAP in astrocytes promotes premature senescence of astrocytes, which further confirmed the senescence-inhibiting effects of YAP in astrocytes [6]. Cyclin-dependent kinase 6 (CDK6), as a downstream molecule of YAP, is decreased in YAP^-/-^ astrocytes *in vivo* and *in vitro*, and over-expression of CDK6 partially rejuvenates YAP^-/-^ astrocytes, indicating that YAP inhibits astrocytic senescence through the CDK6 signaling [6]. Moreover, activation of YAP by XMU-MP-1 (an inhibitor of Hippo kinase MST1/2) improves the cognitive decline of AD model mice [6]. These evidences unravel the positive potential of the YAP-CDK6 pathway in restraining astrocytic senescence in AD (Figure 1).

However, there is one study seems contradictory to us, which shows that decrease of neuron-expressed nuclear YAP is correlated with neuronal necrosis under AD pathology [7]. This study also puts forward that decreased expression of YAP lead to AD, but it emphasizes neuron-expressed YAP, and the consequence of YAP down-regulation is neuronal necrosis, which is contradictory to our results. Nonetheless, previous study has shown that YAP is expressed in astrocytes predominantly, but hardly expressed in neurons [8]. Different samples, brain regions, or different YAP antibodies may explain such a contradiction. Moreover, the expression of YAP and subsequent effects has not been investigated, therefore, the role of YAP and senescent astrocytes in AD could not be exclude, the contribution of neuronal necrosis and astrocytic senescence should be compared, and the causality between neuronal necrosis or astrocytic senescence requires further study. Nevertheless, this study also indicates that YAP is a new target for AD.

In summary, the YAP-CDK6 pathway inhibits astrocytic senescence and prevents cognitive decline during AD. Therefore, YAP may be a novel target for AD.
